# Antisense inhibition of methylenetetrahydrofolate reductase reduces survival of methionine-dependent tumour lines

**DOI:** 10.1038/sj.bjc.6600459

**Published:** 2002-07-02

**Authors:** J Sekhon, P Pereira, N Sabbaghian, A R Schievella, R Rozen

**Affiliations:** Department of Human Genetics, McGill University Health Centre – Montreal Children's Hospital, 4060 Ste. Catherine West, Room 200, Montreal, Quebec H3Z 2Z3, Canada; Department of Pediatrics, McGill University Health Centre – Montreal Children's Hospital, 4060 Ste. Catherine West, Room 200, Montreal, Quebec H3Z 2Z3, Canada; Variagenics Inc., 60 Hampshire Street, Cambridge, Massachusetts, MA 02139, USA

**Keywords:** methionine-dependence, cancer cells, methylenetetrahydrofolate reductase, antisense technology

## Abstract

Transformed cells have been documented to be methionine-dependent, suggesting that inhibition of methionine synthesis might be useful for cancer therapy. Methylenetetrahydrofolate reductase synthesises 5-methyltetrahydrofolate, the methyl donor utilised in methionine synthesis from homocysteine by vitamin B_12_-dependent methionine synthase. We hypothesised that methylenetetrahydrofolate reductase inhibition would affect cell viability through decreased methionine synthesis. Using medium lacking methionine, but containing homocysteine and vitamin B_12_ (M-H+), we found that nontransformed human fibroblasts could maintain growth. In contrast, four transformed cell lines (one colon carcinoma, two neuroblastoma and one breast carcinoma) increased proliferation only slightly in the M-H+ medium. To downregulate methylenetetrahydrofolate reductase expression, two phosphorothioate antisense oligonucleotides, EX5 and 677T, were used to target methylenetetrahydrofolate reductase in the colon carcinoma line SW620; 400 nM of each antisense oligonucleotide decreased cell survival by approximately 80% (*P*<0.01) and 70% (*P*<0.0001), respectively, compared to cell survival after the respective control mismatched oligonucleotide. Western blotting and enzyme assays confirmed that methylenetetrahydrofolate reductase expression was decreased. Two neuroblastoma and two breast carcinoma lines also demonstrated decreased survival following EX5 treatment whereas nontransformed human fibroblasts were not affected. This study suggests that methylenetetrahydrofolate reductase may be required for tumour cell survival and that methylenetetrahydrofolate reductase inhibition should be considered for anti-tumour therapy.

*British Journal of Cancer* (2002) **37**, 225–230. doi:10.1038/sj.bjc.6600459 www.bjcancer.com

© 2002 Cancer Research UK

## 

Folate derivatives participate in single-carbon transfers in several reactions, including the synthesis of nucleotides and methionine. Methionine is the precursor of S-adenosylmethionine which is utilised in numerous transmethylation reactions, including DNA methylation. Methylenetetrahydrofolate reductase (MTHFR) converts 5,10-methylenetetrahydrofolate, the methyl donor in thymidine synthesis, into 5-methyltetrahydrofolate, the predominant circulatory form of folate. 5-Methyltetrahydrofolate is a co-substrate for homocysteine remethylation to methionine by the vitamin B_12_-dependent methionine synthase. Consequently, MTHFR maintains the balance between folates utilised in DNA synthesis and folates utilised in the methionine cycle/DNA methylation. A common variant in MTHFR, an alanine to valine substitution in exon 4, 677C→T (Goyette *et al*, 1994; Frosst *et al*, 1995), has been implicated in many complex disorders. This polymorphism, which is present in the homozygous state in 5–20% of North Americans and Europeans, has been reported to decrease risk of colon cancer (Ma *et al*, 1997) and adult lymphocytic leukaemia (Skibola *et al*, 1999). Homozygosity is associated with a mild enzymatic deficiency (approximately 40% of control values) (Frosst *et al*, 1995).

Identification of biochemical differences between normal and transformed cells may aid in the treatment of human cancers. Methionine auxotrophy of transformed cells is one such biochemical change. This methionine dependence may be due to the high methionine requirement for transmethylation reactions resulting in low free-methionine levels and low S-adenosylmethionine/S-adenosylhomocysteine ratios (Coalson *et al*, 1982; Guo *et al*, 1993a). Many tumour-derived cell lines show sensitivity to a limited methionine supply and arrest in late-S/G2 stage of the cell cycle *in vitro* and *in vivo* (Guo *et al*, 1993a,b; Hoffman and Jacobsen, 1980).

This study examined methionine dependence in nontransformed fibroblasts and in several transformed lines (one colon carcinoma, two neuroblastoma, one breast carcinoma). In a second series of experiments, we downregulated MTHFR, due to its role in methionine synthesis, by transfection of antisense oligonucleotides (ASOs) in order to evaluate survival of the transformed cells. Antisense technology has been successfully employed to downregulate the expression of numerous genes in tumour cells (Crooke, 1992). These ASOs can inhibit gene expression by forming RNA-DNA duplexes resulting in a decrease in the target gene products (Zamecnik, 1996). Data presented in this study demonstrate that antisense-mediated reduction of MTHFR expression is associated with a substantial decrease in cell viability of transformed cells.

## MATERIALS AND METHODS

### Cell lines

Human fibroblasts MCH 51 and MCH 75 were obtained from the Repository for Mutant Human Cell Strains (Montreal Children's Hospital, Montreal, Canada). Human colon carcinoma cell line SW620 and two neuroblastoma lines (BE(2)C and SKNF-1) were obtained from American Type Culture Collection (Rockville, MD, USA). The breast carcinoma cell lines, MCF7 and SKBr3, were a gift from Dr Morag Park (McGill University, Montreal, Canada). Genotyping for the MTHFR variant at bp 677 was performed by PCR amplification and *Hinf*I digestion, as previously reported (Frosst *et al*, 1995). The MCF7 cell line was grown in α-minimal essential medium (α-MEM) (Life Technologies, Rockville, MD, USA) and the SKBr3 cell line was maintained in D-MEM (Life Technologies). Media for both lines was supplemented with 10% foetal bovine serum (Intergen, Purchase, NY, USA). The other cell lines were grown in MEM (Life Technologies) supplemented with 5% foetal bovine serum (Intergen) and 5% iron enriched calf serum (Intergen).

All media was also supplemented with 50 IU ml^−1^ penicillin (Life Technologies), 50 μg ml^−1^ streptomycin (Life Technologies), 0.5 μg ml^−1^ fungizone reagent (Life Technologies). All cell lines were cultured in 75 cm^2^ flasks in a humidified 37°C incubator in 5% CO_2_.

### Deficient culture media

MEM and MEM without folate and methionine supplemented with 100 mM sodium pyruvate (F-M-) were obtained from Life Technologies. For methionine-deficient (M-) media, 2.3 μM folate (Sigma-Aldrich, Oakville, ON, USA) was added to the F-M- media. For all media, 5% foetal bovine serum (Intergen), 5% iron enriched calf serum (Intergen), 50 IU ml^−1^ penicillin (Life Technologies), 50 μg ml^−1^ streptomycin (Life Technologies), and 0.5 μg ml^−1^ fungizone reagent (Life Technologies) were added. For methionone-deficient medium supplemented with homocysteine and vitamin B_12_ (M-H+) media, 0.44 mm DL-homocysteine (Sigma-Aldrich) and 1.5 μM vitamin B_12_ (Sigma-Aldrich) was added to the M- media. Dialysed serum was used for all experiments with deficient media. These conditions were based on similar studies in fibroblasts and in transformed lines (Hoffman and Erbe, 1976; Rosenblatt and Erbe, 1977).

### Cell survival studies of cells in deficient media

Cell viability studies were performed in 6-well tissue culture plates starting with 30 000–50 000 cells per well and three replicates for each condition. The initial number of cells were estimated with a hemocytometer. Cell survival in MEM was used as a control for proliferation in deficient media (M-, M-H+). Surviving cells were counted using the FluoroReporter Colorimetric Cell Protein Assay Kit (Molecular Probes, Eugene, OR, USA).

### Phosphorothioate oligonucleotides

Oligonucleotides (20 bp) with phosphorothioate backbones were obtained from Synthetic Genetics (San Diego, CA, USA) dissolved in 1×Tris-EDTA pH 7.4. The oligonucleotides in this study were: **EX5** (targets nucleotides 796–815 in exon 5 of the human MTHFR mRNA sequence), sequence 5′-AGC TGC CGA AGG GAG TGG TA-3′; **CTSEX5** (control oligonucleotide, scrambled version of EX5 with the same base composition but a randomised sequence); **677T** (targeting nucleotides 668–687 in exon 4 of the human MTHFR mRNA with valine as the polymorphic codon), sequence 5′-GAT GAA ATC GAC TCC CGC AG-3′; **677C** (1 bp mismatch control sequence targeting the alanine allele of human MTHFR), sequence 5′-GAT GAA ATC GGC TCC CGC AG-3′; **CT677** (control oligonucleotide which has six mismatches compared to the sequence of 677T), sequence 5′-AAC GAT AGC GTC TCC CGC AT-3′. The sequences of CT677 and CTSEX5 did not show homology to any known human genes in a BLAST search.

### Transfection with oligonucleotides and cell counting

Cells were plated in 6-well dishes at 50–70% confluence and incubated overnight in complete medium (Life Technologies). Each well was washed once with OPTI-MEM I (Life Technologies). The cells were then overlaid with 1 ml of Opti-MEM I media containing 12 μg ml^−1^ Lipofectin reagent (Life Technologies) per 400 nM of oligonucleotide. The media was replaced with complete media (2–4 ml) after 5 h incubation at 37°C with the ASOs. Transfection with oligonucleotides was performed on 3 consecutive days followed by a 3-day period of regrowth in MEM. Cells were counted by SRB staining as outlined in the FluoroReporter Colorimetric Cell Protein Assay Kit (Molecular Probes). In each experiment, treatments were performed in triplicate.

In dose response experiments, the total oligonucleotide concentration was held constant at 400 nM by supplementing the tested oligonucleotide with the control oligonucleotide (Basilion *et al*, 1999).

### Protein extraction after treatment with oligonucleotides

For Western blot analysis and MTHFR enzyme assays, 6×10^5^ SW620 colon carcinoma cells were plated in 100 mm tissue culture treated petri dishes. After transfection of cells with oligonucleotides, the cells were harvested, and crude protein extracts from cell pellets were obtained by freezing the pellet at −70°C and thawing to 4°C three successive times. The cell pellet was then resuspended in 0.1 M KPO_4_ pH 6.3 with 2 μg ml^−1^ aprotinin (Boehringer Mannheim, Laval, Quebec, Canada) and 2 μg ml^−1^ leupeptin (Amersham Pharmacia Biotech, Piscataway, NJ, USA). Cellular debris was cleared by centrifugation at 14 000 r.p.m. for 10 min. Protein concentration was assayed using the Bradford method (Bradford, 1976) according to the manufacturer's instructions (BioRad, Mississauga, ON, USA).

### MTHFR enzyme assay

Enzyme activity was measured in the reverse direction, in crude protein extracts, as previously described (Christensen *et al*, 1997). Equal amounts of protein (∼60 μg) were used per assay. Enzyme activity was expressed as nmol formaldehyde formed per mg protein h^−1^.

### Western blot analysis

Equal amounts of protein (35–60 μg) were loaded onto a 10% SDS polyacrylamide gel. Transfer was performed in a transfer buffer (39 mM glycine, 49 mM Tris base, 0.037% SDS, 20% methanol) for 2–3 h at 70 V to nitrocellulose (Hybond ECL membrane, Amersham Pharmacia Biotech). The membrane was blocked with 5% non-fat skim milk in PBS-0.5% Tween 20 (Tween 20; BioRad) overnight at 4°C and then cut for separate incubations with antibodies against MTHFR and actin. The MTHFR protein was detected using a rabbit anti-porcine MTHFR antibody (previously-utilised in Frosst *et al*, 1995) at a dilution of 1 : 1000 in 5% non-fat skim milk in PBS-0.5% Tween 20 incubated for 4–6 h at 4°C. The actin antibody was utilised according to the instructions of the supplier (Amersham Pharmacia Biotech). After three successive washes in PBS-0.5% Tween 20, anti-rabbit horseradish peroxidase-conjugated antibody (Amersham Pharmacia Biotech) was used as a secondary antibody. The immunocomplexes were visualised by enhanced chemiluminescence with an ECL kit (Amersham Pharmacia Biotech). Quantitation of protein was determined by scanning the films with a flat-bed scanner (Hewlett Packard Scan). MTHFR has been shown to have two isoforms (Frosst *et al*, 1995). Both MTHFR bands and the actin band areas were calculated; MTHFR protein level is expressed as a ratio of MTHFR/actin.

### Statistical analysis

One-way ANOVA was performed using SPSS software, Version 10.0 (SPSS Inc., Chicago, IL, USA), to analyse cell survival after treatment with EX5. Statistical analysis of cell survival data of SW620 cells following treatment with 677T was performed with the SAS-PC statistical software, Version 8.0 (SAS Institute, Cary, NC, USA). The mixed effects analysis of variance model was used. In this model two factors were considered: drug and concentration. The Student *t*-test was used to evaluate differences in MTHFR activity, and to analyse cell survival data of fibroblast cell lines treated with EX5, 677C and 677T antisense.

## RESULTS

### Growth studies in methionine-deficient media

**Figure 1:**
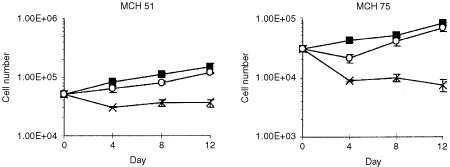
Growth of fibroblast cell lines in deficient media. Two fibroblast cell lines (MCH 51, MCH 75) were grown in MEM (▪), M- (×), and M-H+ (○) for 12 days. The number of cells for each line was counted using the SRB assay at three time points. Each point represents the mean of three replicates±s.d.

**Figure 2:**
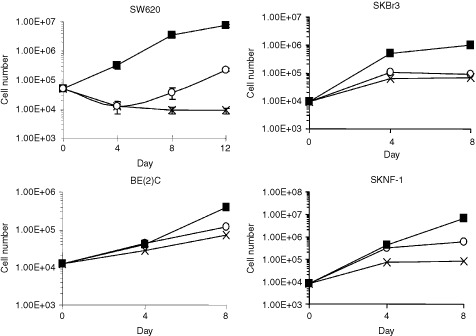
Growth of transformed cell lines in deficient media. The colon carcinoma cell line, SW620, was grown in MEM (▪), M- (×), and M-H+ (○) for 12 days. The number of cells was counted using the Sulfarhodamine B (SRB) assay at three time points. Each point represents the mean of three replicates±s.d. The same experiment was performed for a breast carcinoma line (SKBr3) and two neuroblastoma lines (BE(2)C and SKNF-1), except that the cells were grown for 8 days and each point represents the mean of duplicates.

Two fibroblast strains (Figure 1

) and 4 transformed lines (Figure 2

) were grown in MEM, MEM without methionine (M-), or MEM without methionine supplemented with homocysteine and vitamin B_12_ (M-H+). The latter medium served to examine *de novo* synthesis of methionine from homocysteine and 5-methyltetrahydrofolate, catalysed by vitamin B_12_-dependent methionine synthase. 5-Methyltetrahydrofolate is the product of the MTHFR reaction. All six lines showed sensitivity to the M- medium; growth was significantly reduced in this medium compared to that in MEM. The fibroblasts (MCH 51, MCH 75) could maintain virtually normal growth in the M-H+ medium. However, the transformed lines (colon carcinoma SW620, breast carcinoma SKBr3 and neuroblastomas BE(2)C and SKNF-1) cultured in the M-H+ medium increased their proliferation only slightly through endogenous methionine synthesis (Figure 2). The cell numbers were just a small percentage (5–25%) of the values obtained in MEM. The SKBr3 line was also tested with a lower concentration of homocysteine in the M-H+ medium (0.2 mM DL-homocysteine); the results were similar to those with the higher concentration (0.44 mM) (data not shown).

### Treatment with the EX5 antisense

**Figure 3:**
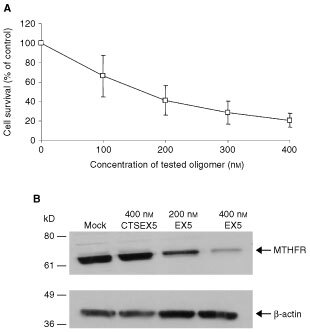
Cell survival and MTHFR protein levels after treatment with the antisense oligonucleotide EX5. (**A**) Colon carcinoma SW620 cells were treated on three successive days with increasing concentration of EX5. Cells were also treated with a control ASO, CTSEX5. The number of surviving cells was determined by SRB staining. Cell survival after transfection with EX5 is expressed as a % of survival after transfection with the control CTSEX5 oligonucleotide. Error bars represent±s.e. of the mean of three experiments, each performed in triplicate. (**B**) MTHFR protein levels after three rounds of treatments with Lipofectin only (mock transfection), 400 nM of CTSEX5, 200 nM of EX5 or 400 nM of EX5. SW620 cells were harvested after the third treatment and subjected to Western blot analysis. The position of the MTHFR protein and the molecular weight markers are indicated. Protein levels of β-actin were also assayed by Western blotting to verify equal loading of samples.

A BLAST search identified sequences in exon 5 of MTHFR that did not have any homology with other ESTs in the NCBI database. Figure 3a

demonstrates a dose-dependent decrease in cell survival (*P*<0.01, one-way ANOVA) after treatment of SW620 carcinoma cells with EX5. At the maximal dose of 400 nM, cell survival decreased approximately 80% compared to that of cells treated with the scrambled control oligonucleotide.

To ensure that MTHFR expression was altered, Western blotting was used to analyse immunoreactive MTHFR protein, after three consecutive treatments with the EX5 ASO. In previous work, we showed that two MTHFR isoforms are present in human tissues (Frosst *et al*, 1995); these isoforms differ only at the N-terminus of the protein encoded by the 5′ end of the mRNA. Consequently, the antisense should inhibit expression of both isoforms. Figure 3b demonstrates a significant decrease in MTHFR protein levels after EX5 treatment, compared to treatment with the scrambled control, CTSEX5, or compared to treatment with Lipofectin reagent only (mock transfection). After normalisation to actin, MTHFR protein levels following treatment with the control oligo were 94% of mock-treated cells, whereas treatment with 200 and 400 nM of EX5 oligo, MTHFR protein levels were 39% and 25%, respectively, of that in mock-treated cells (average of three Western blots).

After treatment with 400 nM of EX5, the two neuroblastoma cell lines (BE(2)C and SKNF-1) showed significant decreases in cell survival compared to control ASO treated cells: decreases of 80% (*P*<0.001) and 65% (*P*<0.01), respectively (data not shown). Similarly, the breast carcinoma cell lines SKBr3 showed a 80% (*P*<0.0001) decrease in cell survival and the MCF7 breast carcinoma line showed a 92% (*P*<0.0001) reduction in cell survival compared to control oligo treated cells (data not shown). Contrary to data obtained in transformed lines, two human fibroblast cell lines (MCH 75 and MCH 51) treated with 400 nM of EX5 did not exhibit significant differences in cell survival compared to control oligo treated cells (*P*>0.05; data not shown).

### Treatment with the 677T antisense

A second oligonucleotide was chosen to target a different region of the mRNA – exon 4, which is the location of the common variant of MTHFR at bp 677. The SW620 line carries the valine allele with a T at bp 677. The 677T antisense should target the valine allele in SW620 whereas the 677C antisense, with one mismatch, should be less effective. The control oligonucleotide, CT677, has a 6 bp mismatch compared to the experimental oligonucleotide.

**Figure 4:**
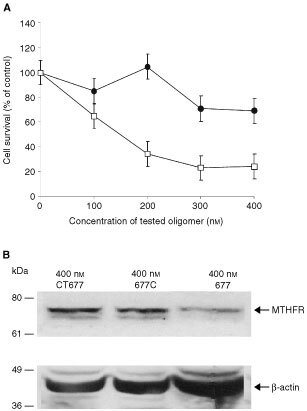
Cell survival and MTHFR protein levels after treatment with the antisense oligonucleotide 677T. (**A**) SW620 cells were treated on 3 successive days with increasing concentrations of 677T (□) or 677C (•). 677C has a single mismatch compared to 677T, at the polymorphic sequence in exon 4. The control oligonucleotide, CT677, contains six mismatches to the 677T sequence. The number of surviving cells was determined by SRB staining. Cell survival after transfection of 677T or 677C is expressed as a percentage of the value obtained with the control CT677 oligonucleotide. Each value on the graph represents the mean±s.e. for five experiments, each performed in triplicate. (**B**) SW620 cells were treated with 400 nM of CT677, 400 nM of 677C, or 400 nM of 677T. Cells were harvested after 24 h of treatment and subjected to Western blot analysis. The position of the MTHFR protein and of the molecular weight markers is indicated. Protein levels of β-actin were also assayed by Western blotting to verify equal loading of samples.

The cell survival of SW620 colon carcinoma cells treated with 677T differs significantly from cells treated with the mismatched control, CT677, at all four tested concentrations between 100 and 400 nM (*P*<0.02 for 100 nM, *P*<0.0001 for 200–400 nM) (Figure 4a

). Cell survival decreased in a dose-dependent manner. At the highest concentration, 400 nM, cell survival was approximately 70% less than that of cells treated with CT677.

Cell survival after transfection with the 677C oligonucleotide, which differs from 677T by a single mismatch, was significantly higher at concentrations of 200–400 nM (*P*<0.01) than that seen with the 677T ASO (Figure 4a). At a concentration of 100 nM, cell survival does not differ significantly between 677C and 677T treated cells (*P*>0.05). Cell survival after treatment with 677C is not significantly different from cells treated with the control CT677 at concentrations of 100 and 200 nM (*P*>0.05), and shows only small but borderline significant differences at 300 and 400 nM (*P*=0.05 and *P*=0.04, respectively). At the maximal dose of 400 nM, cell survival after 677T transfection is decreased by 70% compared to cells treated with the control ASO, whereas survival after 677C transfection was decreased by only 30%.

Western blotting (Figure 4b) demonstrated that MTHFR protein levels were reduced following a 24-h transfection with the 677T oligonucleotide. MTHFR protein levels following transfection of the 677C or 677T were 81% and 17%, respectively, of the values obtained in cells treated with the control CT677 oligo. In addition, we measured enzyme activity after 24 h of treatment with Lipofectin only (mock treatment), cells treated with 400 nM CT677 (control), and cells treated with 400 nM of 677C or 677T (data not shown). Treatment with 400 nM of 677T reduced MTHFR enzymatic activity significantly (*P*<0.01) compared to mock treatment, to values that were approximately 20% (data not shown). Treatment with 400 nM of the CT677 or 400 nM of the 677C ASO, with the 1 bp mismatch to 677T, shows a borderline significant difference in enzymatic activity compared to mock treated cells (*P*=0.02), but the decrease was minimal (86% of mock activity).

One fibroblast cell line (MCH 75) was used to test the effect of treatment with either 677C or 677T on normal methionine-independent cells. This line is heterozygous for the polymorphism at bp 677. Treatment of MCH 75 cells with either 677C or 677T does not significantly affect cell survival compared to control ASO treated cells (*P*>0.05, data not shown).

## DISCUSSION

This study demonstrates, *in vitro*, the potential of using antisense technology directed against MTHFR to decrease the cell viability of methionine-dependent transformed cells. The analysis of cell survival of nontransformed fibroblasts and of several transformed lines cultured in methionine-deficient medium, with and without homocysteine/vitamin B_12_ supplementation, supports earlier reports of the high methionine requirement of transformed lines (Hoffman, 1984); in that review, 23 human lines and 19 animal cell lines were reported to be methionine-dependent. Although normal fibroblast lines showed the ability to restore their growth by restoring endogenous methionine synthesis, the transformed cell lines only exhibited a slight increase in survival under the same conditions. The amount of recovery may in fact be a reflection of the degree of transformation of the colon carcinoma lines (Breillout *et al*, 1990).

By utilising antisense phosphorothioate deoxyoligonucleotides, *in vitro*, to specifically suppress the expression of MTHFR, the cell viability of a methionine-dependent colon carcinoma line was substantially decreased. 677T was able to decrease cell survival by 70% at the maximal dose of 400 nM, while EX5 decreased cell survival by 80% under similar conditions. The 677T ASO appears to exhibit a high specificity for the valine form of MTHFR present in SW620 cells; the 677C ASO, with only a single mismatch (at the polymorphic nucleotide), did not have as great an effect on cell survival. The differences in cell survival between the 677T and 677C oligos were significantly different for concentrations of 200, 300 and 400 nM. These results are consistent with previous reports demonstrating the specificity for one form of a target gene using phosphorothioate oligonucleotides with single base mismatches as controls (Duroux *et al*, 1995; Bennet *et al*, 1996; Basilion *et al*, 1999). Our observations also suggest that allele-specific targeting may be possible for MTHFR. This approach has been proposed as a method for targeting tumour tissues, which often undergo loss of heterozygosity (LOH), without significantly compromising the gene and its product in non-tumour cells (Basilion *et al*, 1999). For genes that have a common variant and are essential for cell survival, the single remaining allele could be targeted *in vivo* in tumour cells that have undergone LOH, whereas the non-tumour tissue, with two alleles, should still retain the activity of the non-targeted allele and, consequently, remain viable. We have recently demonstrated that MTHFR undergoes 15–20% LOH in colorectal tumours (Pereira *et al*, 1999); LOH for MTHFR may be as high as 45–50% in ovarian tumours (Viel *et al*, 1997).

Treatment of two neuroblastoma cell lines and two breast carcinoma cell lines showed significant decreases in cell survival after treatment with 400 nM of EX5 compared to control oligo treated cells. These results point to the applicability of downregulating MTHFR expression in various types of tumour lines in order to decrease cell survival. To test the effects of targeting MTHFR in nontransformed cells, two human fibroblast cell lines (MCH 75 and MCH 51) were treated. Both cell lines did not show significant changes in cell survival after treatment with 400 nM of EX5, compared to control ASO treated cells. Similarly, treatment of the MCH 75 fibroblast line with 677T or 677C did not significantly affect cell survival, compared to CT677 treated cells. These results suggest that antisense inhibition of MTHFR may have a more deleterious effect on methionine-dependent tumour cells.

The downregulation of MTHFR in methionine-dependent tumour lines may cause decreases in methionine levels to an extent that significantly affects cell survival. The same decreases in methionine production in methionine-independent lines may not substantially affect cell survival since these cell lines are not reliant on such high levels of methionine to sustain cell growth.

ASOs are designed to affect the information transfer from gene to protein, by altering the metabolism of RNA (Crooke, 1999). The end result is expected to be a decrease in levels of the gene product. We therefore examined MTHFR protein levels after antisense treatment. For the EX5 experiments, we performed Western blotting under the same conditions that were used for analysis of cell numbers i.e. following three rounds of transfection. For the 677T experiments, we found that the nonspecific toxicity of the phosphorothioate oligonucleotides was quite high; the toxicity of these oligos has been well documented (Stein and Cheng, 1993; Stein, 1999). Due to the greater toxicity of the 677 oligos (control and experimental ASOs), cell survival was considerably reduced after three rounds of transfection, even though the specific oligo was always associated with lower cell numbers than the control oligo. Consequently, we used one 24-h transfection, rather than three consecutive transfections, to generate enough cells for Western blotting. The conclusions from both sets of Westerns (EX5 and 677T oligos) were similar – MTHFR protein was significantly reduced compared to that of the control oligonucleotides at the studied concentration (400 nM). MTHFR enzyme activity was also measured following the 677T transfection; this experiment demonstrated that the 677T oligo had a direct effect on MTHFR activity.

Though reducing MTHFR expression to decrease cancer cell survival is a novel hypothesis, other groups have successfully used different means to decrease methionine availability for transformed cells. Guo *et al* (1993a) deprived Yoshida sarcoma-bearing nude mice of dietary methionine resulting in tumour regression and extended survival of the mice. Other investigators have used the enzyme methioninase *in vivo* (Tan *et al*, 1996a); this enzyme from *Pseudomonas putida* catalyses the conversion of methionine to methanethiol (Weimer *et al*, 1999). It has been shown to deplete circulating methionine levels in mice and in humans (Lishko *et al*, 1993; Tan *et al*, 1996b). *In vivo* studies injecting purified methioninase into nude mice bearing either rodent or human tumours have successfully arrested the growth of these methionine-dependent tumours with no apparent toxic side effects (Tan *et al*, 1996a).

This study has shown that a reduction in MTHFR levels reduces cell viability *in vitro*. To evaluate the validity of this approach for chemotherapy, these techniques need to be transferred to an *in vivo* model. Inhibition of MTHFR through antisense technology or through other means, such as pharmaceutical agents, should be considered alone or in conjunction with other antifolate compounds, such as methotrexate or 5-fluorouracil, to increase our arsenal of chemotherapeutic reagents.
